# Dynamic Simulation of Nano-Gel Microspheres for Plugging Preferential Flow Channels and Enhancing Oil Recovery in Waterflooded Reservoirs

**DOI:** 10.3390/gels11070536

**Published:** 2025-07-10

**Authors:** Long Ren, Cong Zhao, Jian Sun, Cheng Jing, Haitao Bai, Qingqing Li, Xin Ma

**Affiliations:** 1 School of Petroleum Engineering, Xi’an Shiyou University, Xi’an 710065, China; xjkelsj@163.com (J.S.); jich.0704@163.com (C.J.);; 2 Shaanxi Key Laboratory of Advanced Stimulation Technology for Oi & Gas Reservoirs, Xi’an 710065, China

**Keywords:** nano-gel microspheres (NGMs), preferential flow channels (PFCs), plugging mechanism, numerical simulation, enhanced oil recovery (EOR)

## Abstract

This study addresses the unclear mechanisms by which preferential flow channels (PFCs), formed during long-term waterflooding, affect nano-gel microsphere (NGM) flooding efficiency, utilizing CMG reservoir numerical simulation software. A dynamic evolution model of PFCs was established by coupling CROCKTAB (stress–porosity hysteresis) and CROCKTABW (water saturation-driven permeability evolution), and the deep flooding mechanism of NGMs (based on their gel properties such as swelling, elastic deformation, and adsorption, and characterized by a “plugging-migration-replugging” process) was integrated. The results demonstrate that neglecting PFCs overestimates recovery by 8.7%, while NGMs reduce permeability by 33% (from 12 to 8 mD) in high-conductivity zones via “bridge-plug-filter cake” structures, diverting flow to low-permeability layers (+33% permeability, from 4.5 to 6 mD). Field application in a Chang 6 tight reservoir (permeability variation coefficient 0.82) confirms a >10-year effective period with 0.84% incremental recovery (from 7.31% to 8.15%) and favorable economics (ROI ≈ 10:1), providing a theoretical and engineering framework for gel-based conformance control in analogous reservoirs.

## 1. Introduction

In oilfield development, particularly during the late stages of waterflooding, the formation of preferential flow channels (PFCs) between injection and production wells presents a central challenge to enhancing reservoir recovery [1,2]. As reported by Dai et al. [3] and others, prolonged water injection and reservoir development exacerbate heterogeneity and facilitate the gradual formation of high-conductivity flow channels. These PFCs cause injected water to bypass substantial volumes of residual oil, significantly reducing waterflood efficiency. This triggers the characteristic contradiction of a sudden decline in oil production accompanied by a surge in water production—a phenomenon widely documented in mature oilfields globally [4,5,6].

In recent years, nano-gel microsphere (NGM) modulation drive and dissection technology has shown significant potential for application. Feng et al. [7] experimentally demonstrated, through physical simulations, that nano-elastic microspheres effectively block high-conductivity channels via physical retention and elastic deformation. Combining NMR technology, they observed a transport characteristic in porous media where microspheres migrate through large pores but are retained in small pores, thereby enabling water cut control. Using environmental scanning electron microscopy (ESEM), Zhang et al. [8] revealed significant swelling characteristics in polymer-based NGMs, with swelling ratios reaching up to 15-times. Through microscopic displacement experiments, they observed the formation of a composite blocking structure—comprising bridge plugging and filter cake mechanisms—within pore throats, attributed to mechanical retention. These findings verify the formation of a composite migration–plugging–filter cake blocking structure within pore throats and corroborate the dynamic regulation mechanism characterized by sequential migration, plugging, breakthrough, and replugging [9,10,11,12].

In the field of numerical simulation, scholars are continuously improving relevant models. For instance, Su et al. [13] established a multi-physics coupled model considering microsphere expansion, migration, and plugging characteristics. By incorporating pore structure parameters and fluid–solid interaction characteristics, they achieved the quantitative characterization of microsphere transport behavior. However, key technical bottlenecks persist in existing studies. Firstly, numerical models often fail to accurately characterize the formation mechanism and dynamic evolution process of PFCs. Secondly, the coupled simulation methodology for the plugging mechanism of nanospheres in high-permeability channels and the resulting fluid diversion effect remains underdeveloped.

Building upon this foundation, this study integrates reservoir engineering theory with CMG numerical simulation technology. Through the coupling of the key parameters of CROCKTAB (stress–porosity hysteresis) and CROCKTABW (water saturation-driven permeability evolution), a multi-field coupling model of PFCs and nanospheres is constructed. This model enables the systematic analysis of the evolution of reservoir properties and their influence on development indicators. The research findings aim to provide a technical solution possessing both theoretical value and engineering applicability for enhancing recovery in long-term waterflooded reservoirs.

## 2. Results and Discussion

### 2.1. Numerical Simulation of PFCs in Waterflooding Development

To mitigate computational expense and convergence challenges, a one-injection and one-production well-pair model was adopted. The grid dimensions are 150 × 150 × 2, with planar grid spacing of 1 m × 1 m and vertical spacing of 5 m. The injector–producer distance is 150 m. Key model parameters are detailed in Table 1.

#### 2.1.1. Coupled Simulation Setup for PFC Evolution

**Figure 1:**
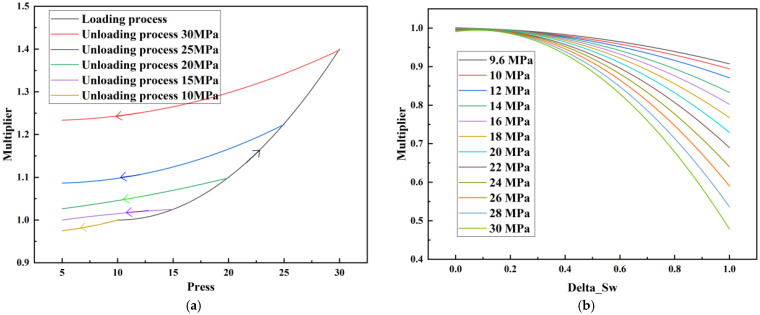
Multiplier effects of pressure (CROCKTAB) and water saturation differential (CROCKTABW). (**a**) CROCKTAB parameterization; (**b**) CROCKTABW parameterization.

CROCKTAB (stress–porosity hysteresis) and CROCKTABW (water saturation-driven permeability evolution) keywords were implemented to simulate PFC formation during waterflooding. CROCKTAB defines pressure-dependent rock compressibility/expansion factors, while CROCKTABW governs pore volume variation as a function of water saturation differentials under varying pressures. The parameterization schemes are illustrated in Figure 1.

(1) CROCKTAB parameters (see Figure 1a) reveal nonlinear porosity growth during loading (black curve), with pronounced hysteresis during unloading. The elastic recovery capacity decays significantly under high-pressure unloading, confirming irreversible deformation from prolonged water injection.
(2) CROCKTABW parameters (see Figure 1b) demonstrate a positive correlation between pore volume expansion and water saturation increments. High-stress conditions (e.g., 30 MPa) intensify pore structure adjustment during aqueous-phase seepage, quantitatively supporting PFC evolution.

#### 2.1.2. Dynamic Evolution of Reservoir Properties During Waterflooding

Integrated CROCKTAB and CROCKTABW simulations reveal distinct property evolution in high-permeability and low-permeability layers (Table 2, Table 3 and Table 4).

Table 2 documents reservoir property evolution during waterflooding from initial development to the 95% water-cut stage. The high-permeability layer exhibits progressive porosity enhancement (initial: from 0.12 to 0.16) and permeability increases (from 8 to 12 mD), demonstrating a linear permeability enhancement trend along the injector–producer alignment. This trend arises from prolonged aqueous-phase scouring that enlarges pore throats and diminishes rock compaction effects, consistent with the effective stress–porosity coupling captured by the CROCKTAB and CROCKTABW models. Concurrently, oil saturation declines sharply from 0.45 to 0.30, forming a distinct low oil-saturation zone between wells. This spatial distribution directly manifests injected-water bypassing due to PFCs, confirming the reservoir heterogeneity challenge characterized by high-permeability channeling.

Table 3 reveals distinct property evolution in the low-permeability layer compared to the high-permeability layer (Table 2). Porosity increased modestly (from 0.08 to 0.10) and permeability rose to 4.5 mD from 3 mD—only one-third the magnitude of the high-permeability layer’s enhancement (12 mD from 8 mD). This muted response stems from injected-water channeling in high-permeability zones, which impedes energy replenishment and delays pore-pressure transmission in low-permeability strata, empirically validating the “high-permeability layer shielding effect.” Oil saturation stabilizes at 0.35 post-decline—significantly exceeding the high-permeability layer’s 0.30 (Table 2)—confirming residual oil enrichment during waterflooding. Limited flow capacity prevents effective exploitation of this resource, establishing the targetable reservoir volume for subsequent NGM flooding through selective fluid diversion.

Table 4 reveals that the longitudinal profile along the injector–producer connection line exhibits a distinct strip-shaped high-permeability zone in the high-permeability layer, accompanied by a corresponding funnel-shaped low oil-saturation zone. This pattern confirms the formation of a highly connected PFC between the wells, consistent with literature findings that long-term water injection exacerbates reservoir heterogeneity.

**Figure 2:**
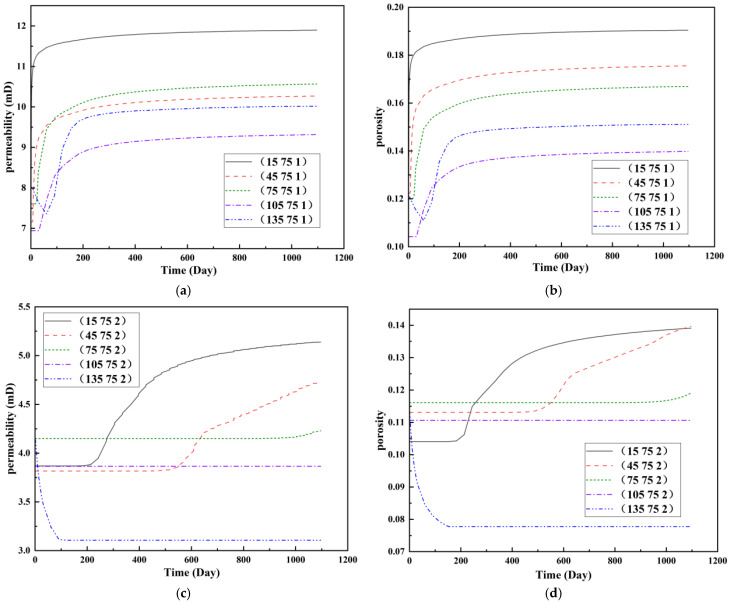
Temporal evolution of porosity and permeability in grid cells of different layers. (**a**) Permeability in high-permeability layer; (**b**) Porosity in high-permeability layer; (**c**) Permeability in low-permeability layer; (**d**) Porosity in low-permeability layer.

To visualize the temporal evolution of porosity and permeability governed by the CROCKTAB and CROCKTABW keywords, specific grid cells were selected for monitoring: five in the high-permeability layer ((15, 75, 1), (45, 75, 1), (75, 75, 1), (105, 75, 1), (135, 75, 1)) and five corresponding grid cells in the low-permeability layer ((15, 75, 2), (45, 75, 2), (75, 75, 2), (105, 75, 2), (135, 75, 2)). The resulting property changes are presented in Figure 2.

Figure 2a,b demonstrates that permeability in the high-permeability layer grids exhibits non-linear growth with water injection time. Notably, permeability near the injection well (e.g., grid (15, 75, 1)) increases from 8 mD to 12 mD within 300 days. In contrast, the grid farthest from the injector (135, 75, 1) requires 600 days to achieve the same increase, confirming the “pressure conduction timeliness”, as injected water preferentially channels through high-permeability pathways. This observation aligns with the effective stress–permeability hysteresis relationship defined by the CROCKTAB keyword. A transient 20% permeability decrease occurred initially in grid (135, 75, 1) due to the pore pressure near the producer falling below the formation pressure, inducing elastic rock expansion. Subsequent pressure recovery from water injection reversed this trend, allowing the permeability to rebound and continue growing.

Porosity in the high-permeability layer also increased from 0.12 to 0.16, displaying a gradient growth trend along the injector–producer direction. The porosity growth rate at grid (15, 75, 1) reached 0.01 per 100 days, while grid (105, 75, 1), situated at the edge of the primary seepage channel, exhibited a significantly lower rate of only 0.005 per 100 days. This verifies the mechanism whereby “the intensity of water flow scouring governs the pore-throat expansion rate”. The porosity evolution in grid (135, 75, 1) closely mirrored its permeability trend.

Figure 2c,d reveals that permeability in the low-permeability layer increased only modestly from 3 mD to 4.5 mD, representing just 50% of the amplitude observed in the high-permeability layer. Proximity to the injection well enabled grid (15, 75, 2) to initiate a slow permeability increase after 600 days of injection. Conversely, grid (135, 75, 2), persistently located in a low-pressure zone, maintained permeability below 4 mD, reflecting the “seepage shielding effect” imposed by the high-permeability layer. Porosity remained constrained between 0.08 and 0.10, substantially lower than in the high-permeability layer. Grid (105, 75, 2), largely unaffected by direct injected water flow, showed negligible porosity change. This confirms the “physical property inertia” characteristic of the low-permeability layer during waterflooding, providing the physical basis for subsequent residual oil enrichment targeted by NGM flooding.

#### 2.1.3. Impact of PFCs on Waterflooding Performance

**Figure 3:**
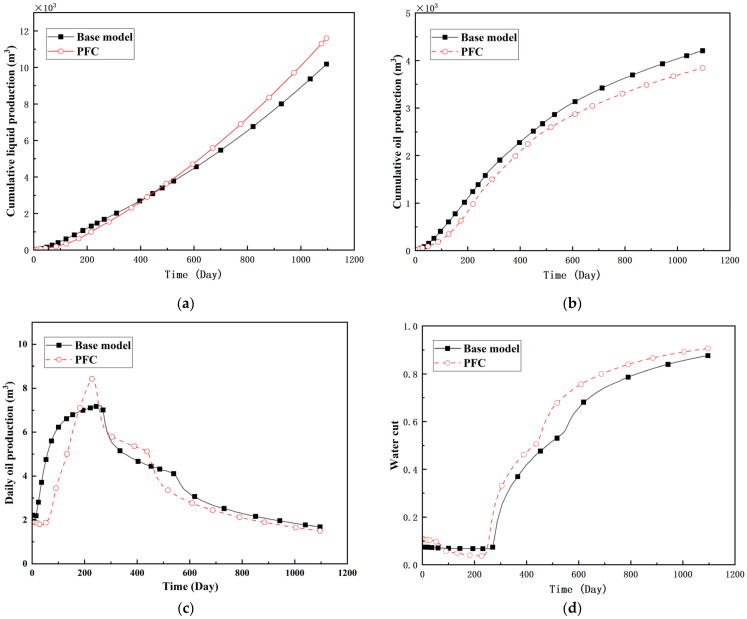
Impact of PFCs on waterflooding performance. (**a**) Cumulative liquid production; (**b**) Cumulative oil production; (**c**) Daily oil production; (**d**) Water cut.

To quantify the macroscopic impact of PFCs on waterflooding efficiency, a 3-year simulation was conducted. The comparative results between the model incorporating PFC evolution (dominant channel model) and the base model (neglecting PFCs) are presented in Figure 3.

The emergence and evolution of PFCs, rigorously captured through coupled geomechanical–fluid flow modeling (CROCKTAB and CROCKTABW), drive fundamentally distinct flooding regimes, with profound implications for recovery efficiency. Figure 3 provides conclusive evidence through four interlinked responses. (1) Early-stage flow divergence vs. late-stage channelization (see Figure 3a): Initial suppression of cumulative liquid production (<500 days) transitions to a 13.85% surplus (11,596 m^3^ vs. 10,185 m^3^) post-PFC maturation. This inversion confirms progressive flow conductivity enhancement as injected water increasingly bypasses low-permeability strata through established high-k pathways. (2) Sweep efficiency deterioration (see Figure 3b): Cumulative oil production decreases by 8.7% (3842 m^3^ vs. 4207 m^3^) due to volumetric sweep reduction, where PFCs sequester >35% of movable oil in unswept low-k zones. (3) Accelerated production decline (see Figure 3c): Post-600 days, daily oil production stabilizes at 15% lower (2.5 m^3^/d) under the “short-circuit effect”—a direct consequence of rapid fluid migration through high-k conduits, while the base model overestimates recovery by neglecting the PFC-induced shielding of low-k contributions. (4) Premature water breakthrough (see Figure 3d): Water cut rises 30 days earlier and plateaus 4% higher (92% vs. 88%), spatially correlating with the low oil-saturation corridor in Figure 2. This behavior validates CROCKTABW’s critical feedback mechanism: incremental water saturation → permeability amplification → accelerated channelization.

### 2.2. Simulation and Application of NGM Flooding After Waterflooding

#### 2.2.1. Mechanism of NGM Profile Control

The numerical simulation technique for NGM profile control is based on three synergistic mechanisms:

(1) NGMs block PFCs through physical retention and elastic deformation, redirecting injected water into low-permeability zones to mobilize residual oil [7].
(2) NGM injection increases the displacing-phase viscosity, enabling viscous forces to surpass capillary thresholds and mobilize trapped crude oil from pore throats [14,15,16].
(3) NGMs’ adsorption reduces oil-wetting sites on rock surfaces, shifting wettability toward water-wet states (validated by ESEM studies [8]). This decreases contact angles, reducing capillary forces that trap residual oil.

**Figure 4:**
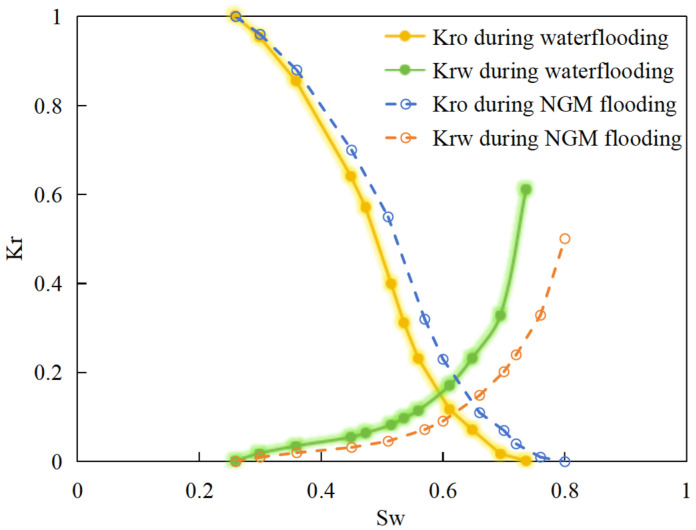
Influence of NGM flooding on oil-water relative permeability curves.

Consequently, oil-phase relative permeability *K*_ro_ increases since weakened oil–rock adhesion improves oil mobility, while water-phase relative permeability *K*_rw_ decreases as adsorbed NGMs constrict pore throats and increase flow resistance (Figure 4).

Figure 4 illustrates alterations in the phase-relative-permeability curves before and after NGM displacement. The yellow and green solid lines represent these parameters during waterflooding, whereas blue and orange dashed lines denote oil-phase and water-phase relative permeability during NGM displacement after waterflooding, explicitly showing the *K*_ro_ increase and *K*_rw_ decrease described above.

Comparing the relative permeability curves of waterflooding and NGM flooding shows different features. In the early stage (*S*_w_ 0~0.3), *K*_ro_ values are very low and decrease slowly, while *K*_rw_ values remain low with minimal increases. This indicates that at low water saturation, water exists primarily in small pores or in a dispersed state with limited flow capacity, while oil dominates the pore channels. Consequently, low *S*_w_ increases have a minimal impact on oil-phase permeability. In the middle stage (*S*_w_ 0.3~0.6), increasing *S*_w_ causes more significant Krw increases as water occupies the oil-phase pore channels, restricting oil flow. In the late stage (*S*_w_ > 0.6), the initial Krw rises faster than the NGM *K*_rw_, indicating that NGMs alter water-phase viscosity and increase flow resistance. The NGM-phase relative permeability curve resembles the oil–water curve, but post-NGM flooding, oil-phase relative permeability end values increase and residual oil saturation decreases. The rightward shift of the iso-saturation point (from 0.59 to 0.65) further confirms enhanced oil mobility. The water-phase relative permeability at residual oil saturation decreased by 0.109 (from 0.61 to 0.501). Post-nano-fluid flooding-phase permeability curve changes resulted from nanoparticle adsorption-induced wettability reversal in the porous medium. The reduction in oil-wetting sites due to NGM adsorption shifts rock wettability toward water-wet states, decreasing the contact angle and capillary trapping forces. At high water saturation stages (Sw = 0.65), this elevates the oil-phase relative permeability (Kro) by 0.07 (from 0.07 to 0.14) and suppresses the water-phase relative permeability (Krw) by 0.09 (from 0.23 to 0.14).

Collectively, these mechanisms mobilize crude oil trapped within capillaries from pore throats, displacing residual oil and reducing phase relative permeability residual oil saturation. The calculation formula for the reduction value of residual oil saturation by NGMs is as follows:

(1) $$\Delta S_{\mathrm{or}}=\alpha \cdot C_{\mathrm{ads}}(S_{\mathrm{ori}}-S_{\mathrm{orE}})$$

where ∆*S*_or_ denotes the reduction value of residual oil saturation; *α* is the calibrated coefficient from coreflood experiments; *C*_ads_ denotes the NGM adsorption concentration; *S*_ori_ represents the initial residual oil saturation in the waterflooding stage; and *S*_orE_ indicates the water-driven residual oil saturation in the EOR stage.

The dynamic water-phase endpoint permeability is scaled by the NGM adsorption concentration *C*_ads_, as follows:

(2) $$K_{\mathrm{rw}}^{\mathrm{end}}=K_{\mathrm{rwi}}^{\mathrm{end}}(1-\beta \cdot C_{\mathrm{ads}})$$

where $K_{rw}^{end}$ represents the dynamic water-phase endpoint permeability in NGM flooding; $K_{rwi}^{end}$ represents the initial water-phase endpoint permeability in the waterflooding stage; and *β* is calibrated coefficient from coreflood experiments.

Relative permeability curves are dynamically adjusted using CMG’s ADSCOMP and ADSTABLE keyword, linking *C*_ads_ to grid-block properties in the dynamic effective permeability calculation formula. This method integrates geomechanical effects (CROCKTAB and CROCKTABW) with chemical flooding responses (ADSCOMP and ADSTABLE), enabling the full coupling of wettability dynamics.

NGMs’ properties critically enable profile control; significant swelling (up to 15×) allows 3~6× in-situ expansion, forming essential “bridge-plug and filter-cake” structures for PFC blockage. Concurrently, pore-surface adsorption reduces effective pore radii, while elastic deformation facilitates pore-throat migration and structural adaptation. These mechanisms collectively enhance PFC plugging and oil recovery.

#### 2.2.2. Simulation of NGM Flooding Effects

Following the establishment of PFCs in the high-permeability layer at waterflooding termination (Section 2.1.2), a 1% NGM suspension was injected. The NGMs comprised environmentally friendly polyacrylamide/cellulose composite gels, nano-modified for enhanced thermal stability (>90 °C) and salinity tolerance (>20,000 ppm). Based on these stability thresholds, NGMs were assumed to maintain stable gel properties throughout the 10-year simulation period, though degradation effects will be incorporated in future studies as experimental data becomes available. This formulation renders them suitable for low-permeability/fractured reservoirs. Key property evolution patterns are quantified in Table 5, Table 6 and Table 7.

High-permeability layer dynamics (Table 5): Permeability decreased abruptly from 12 mD to 8 mD post-injection, followed by gradual recovery to 10 mD over 2000 days. This reduction arose from microsphere-induced pore-throat blockage via a “bridge-plug and filter-cake” mechanism, elevating the displacement pressure—consistent with the inverse pressure–permeability correlation in the CROCKTABW model (Section 2.2). Partial permeability recovery after microsphere breakthrough reflected “dynamic plugging-rebreakthrough” behavior, though values remained below initial waterflooding levels. Porosity concurrently declined from 0.16 to 0.14, confirming that NGM retention reduced the effective pore-throat volume. Oil saturation rebounded from 0.30 to 0.35, indicating fluid diversion to low-permeability zones and secondary displacement of residual oil.

Low-permeability layer response (Table 6): Permeability increased by 33% (from 4.5 mD to 6.0 mD) after a 500-day lag—attributable to preferential NGM plugging of high-conductivity pathways. This delayed enhancement aligns with the “block-before-diversion” fluid-flow principle. Near-injector grids (e.g., (15, 75, 2)) exhibited a permeability growth rate of 0.003 mD/100 days, doubling the waterflooding rate (0.0015 mD/100 days), validating the diversion efficacy. Oil saturation declined from 0.35 to 0.28, demonstrating residual oil mobilization. The 40% acceleration in oil-saturation decline relative to waterflooding confirmed NGMs’ enhanced recovery via seepage-field reconfiguration.

Longitudinal profile evolution (Table 7): The injector–producer connection line transitioned from a “strip-shaped high-permeability zone” (Section 4.2, Table 4) to a “dumbbell-shaped low-permeability zone”. Permeability decreased by 30% in central high-k regions while increasing by 20% at low-k peripheries, with the seepage-direction deflection reaching 45°. This reconstructed flow field elevated the inter-well pressure gradient from 0.02 MPa/m to 0.035 MPa/m, improving driving-energy distribution uniformity.

**Figure 5:**
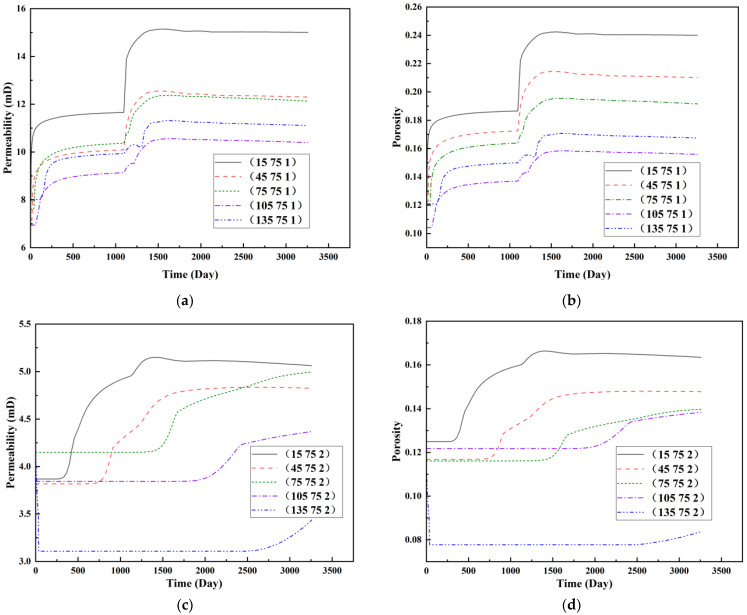
Evolution of porosity and permeability after NGM flooding. (**a**) Permeability of high-permeability layer grids; (**b**) Porosity of high-permeability layer grids; (**c**) Permeability of low-permeability layer grids; (**d**) Porosity of low-permeability layer grids.

To visualize the plugging effect of NGMs on large pore throats and PFCs, five grids in the high-permeability layer ((15, 75, 1), (45, 75, 1), (75, 75, 1), (105, 75, 1), (135, 75, 1)) and five grids in the low-permeability layer ((15, 75, 2), (45, 75, 2), (75, 75, 2), (105, 75, 2), (135, 75, 2)) were selected to monitor changes in permeability and porosity. The resulting property evolution curves during the entire waterflooding and NGM flooding cycle are shown in Figure 5.

Comprehensive analysis of Figure 5a,b reveals that following NGM injection, porosity and permeability trends in high-permeability grids exhibit an initial rapid increase followed by a gradual decline. This behavior arises because NGM blockage of large pore throats elevates the displacement pressure, temporarily enhancing the porosity and permeability. Subsequent NGM breakthrough reduces the pore pressure, causing corresponding property declines. Locally, NGM-driven changes align with trends observed during pure waterflooding, with grids near injector and producer wells showing accelerated pressure responses. Critically, porosity fluctuations in grid (135, 75, 1) are attributable not to near-wellbore pressure-induced rock deformation, but to NGM plugging and breakthrough dynamics within pore throats.

Analysis of Figure 5c,d indicates that grids (15, 75, 2) and (45, 75, 2) in the low-permeability layer display similar NGM-induced trends. However, reduced initial water influx into this layer mitigates property fluctuations compared to high-permeability zones. Furthermore, while NGMs block major flow channels, inferior reservoir properties promote radial propagation of injected fluids and pressure. Consequently, grids (75, 75, 2), (105, 75, 2), and (135, 75, 2) exhibit delayed pressure responses without distinct decline phases.

#### 2.2.3. Macro-Scale Impact of NGMs on Oil Recovery

**Figure 6:**
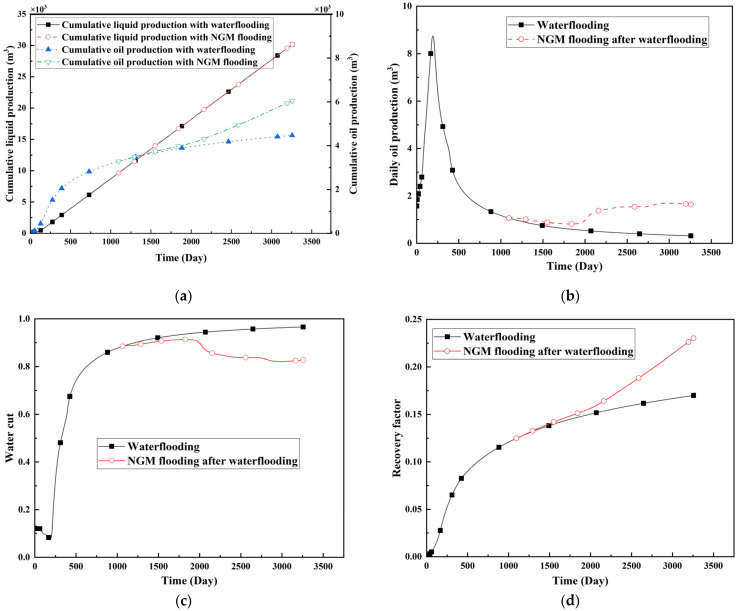
Macro-scale impacts of NGMs on production performance. (**a**) Cumulative liquid and oil production; (**b**) Daily oil production; (**c**) Water cut; (**d**) Recovery factor.

To quantitatively evaluate the macro-scale impact of NGMs on reservoir development efficiency, Figure 6 presents comparative simulation results of production dynamics for two development scenarios: continuous waterflooding versus NGM flooding initiated after high water-cut (90%) in production wells.

Comparative analysis of development schemes reveals that NGMs significantly enhance recovery efficiency. Under equivalent liquid production volumes, NGM implementation increases cumulative oil production by 7000 m^3^ and elevates ultimate recovery by 0.84 percentage points. This improvement stems from NGMs’ “bridge-plug-filter cake” mechanism obstructing high-permeability channels, forcing fluid diversion into previously under-swept low-permeability zones.

The modest early-stage oil production increase reflects initial PFC plugging and residual oil mobilization in waterflooded high-permeability regions. A critical inflection point occurs at 2096 days (1000 days post-NGM injection), where cumulative oil production curves diverge sharply and daily production surges. This correlates with permeability enhancement in low-permeability layers (Section 2.2.2, Table 6), confirming the “plug-before-divert” mechanism. Radial flow propagation—induced by PFC blockage and exacerbated by inferior reservoir properties in low-permeability zones—delays pressure transmission to peripheral grids (e.g., (75,75,2), (105,75,2), and (135,75,2)), explaining their attenuated property responses.

### 2.3. Field Application: Case Study of the Chang 6 Tight Oil Reservoir

**Figure 7:**
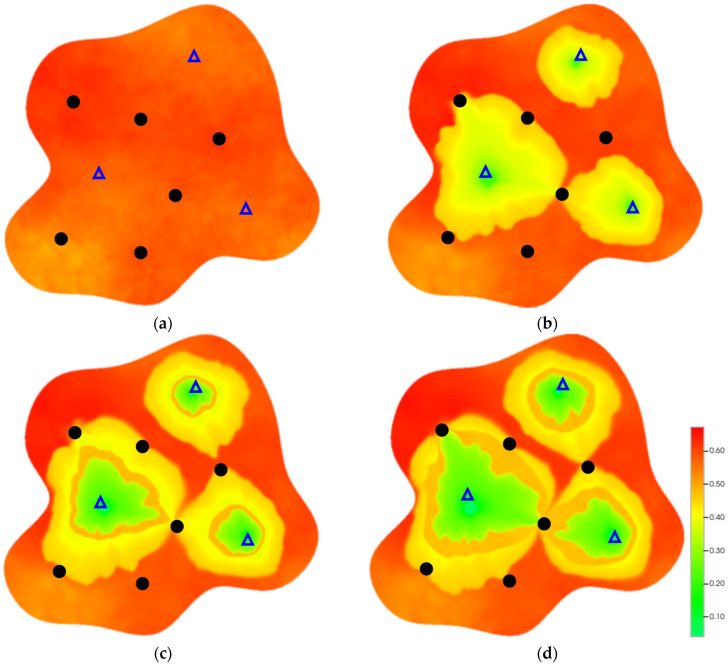
Full-cycle evolution of oil saturation fields under waterflooding and NGM flooding in Chang 6 Reservoir (Triangles represent injector wells, circles represent producer wells). (**a**) Initial stage; (**b**) End of waterflooding; (**c**) 6 years post-NGM injection; (**d**) 12 years post-NGM injection.

**Figure 8:**
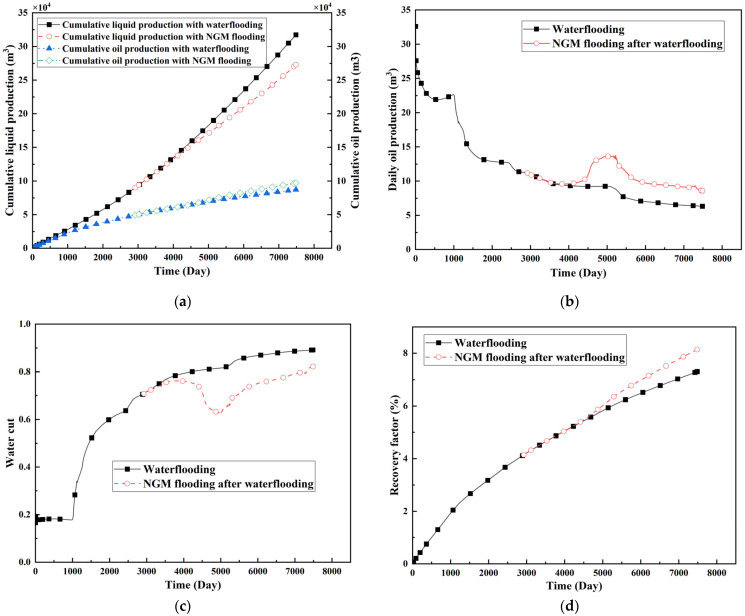
Production dynamics of Chang 6 Reservoir under different development schemes. (**a**) Cumulative liquid and oil production; (**b**) Daily oil production; (**c**) Water cut; (**d**) Recovery factor.

The Jiyuan Oilfield, primarily comprising lithologic and structural–lithologic composite reservoirs, hosts the Chang 6 tight oil reservoir. This reservoir exhibits pronounced lateral and vertical heterogeneity (with a permeability variation coefficient of 0.82), characterized by directional permeability anisotropy from natural fractures (*K*_x_/*K*_y_ = 8), tight lithology and complex oil–water relationships. A numerical simulation model, incorporating a three-injector and six-producer well pattern based on actual geological parameters, was constructed to represent this challenging reservoir. The pronounced heterogeneity accelerates the PFCs during waterflooding, manifested by a rapid water cut rise to 92%, which severely constrained the contribution of liquid production to recovery. To investigate the impact of NGMs on reservoir performance and recovery factor under these complex conditions, NGMs were introduced after the initial waterflooding period. The simulation spanned a total of 20 years. The corresponding results are presented in Figure 7 and Figure 8.

Comparison of the oil saturation fields across different time periods reveals that during the initial 8 years of waterflooding, injected water reached the production wells, establishing PFCs between the injectors and producers. Following NGM injection, an oil bank forms around the injection well and subsequently propagates towards the production wells. Furthermore, the residual oil saturation following NGM flooding is demonstrably lower than that observed after conventional waterflooding.

Analysis of production parameters for the different development schemes indicates that cumulative liquid production was higher for the scenario without NGMs compared to that with NGMs, whereas cumulative oil production exhibited the opposite trend. This demonstrates that NGMs effectively block the PFCs formed during long-term waterflooding development, thereby diverting injected water to displace residual oil from smaller pores. NGM flooding exhibits a significant response lag; the cumulative oil production curves diverge markedly at approximately 4000 days, coinciding with an increase in daily oil production. Following the divergence point, daily oil production under NGM flooding rapidly increased to 17 m^3^/d before gradually declining to 8 m^3^/d; however, it consistently exceeded the production rate achieved without NGMs. Simultaneously, the results indicate that the effective period of incremental oil production attributable to NGMs exceeded ten years, accompanied by a significant reduction in water cut for the simulated reservoir block. At the conclusion of the 20-year simulation period, the recovery factor reached 7.31% without NGMs and 8.15% with NGMs, representing an increase of 0.84 percentage points. At a conservative oil price of 70/bbl USD, preliminary economic assessment confirms a favorable ROI ≈ 10:1 for NGM flooding in the Chang 6 reservoir, where incremental revenue from the 0.84% recovery gain offsets chemical and operational costs, with key economic drivers being the NGM unit price, heterogeneity severity, and treatment lifetime.

## 3. Conclusions

This study establishes an integrated fluid–solid coupling model for PFCs and NGMs using CMG numerical simulation, systematically revealing the synergistic mechanisms of NGM plugging and EOR. Key conclusions are drawn as follows:

(1) Dynamic quantification of PFC evolution was achieved through coupled CROCKTAB (stress–porosity hysteresis) and CROCKTABW (water saturation-driven permeability evolution) modeling, revealing that conventional simulations overestimate recovery by 8.7% due to unaccounted permeability growth (from 8 to 12 mD, +50%) in high-conductivity layers during long-term waterflooding.
(2) NGMs enable effective conformance control via synergistic “bridge-plug-filter cake” structures leveraging gel properties (15× swelling ratio, elasticity, adsorption), reducing thief-zone permeability by 33% (from 12 to 8 mD) while enhancing low-permeability layer flow capacity by 33% (from 4.5 to 6 mD) and decreasing residual oil saturation (from 0.35 to 0.28).
(3) Field validation in the Chang 6 tight reservoir (permeability variation coefficient 0.82) confirmed a >10-year sustained performance: 0.84% incremental recovery (from 7.31% to 8.15%) with 8% water-cut reduction and favorable economics (ROI ≈ 10:1 at 70/bbl USD), demonstrating engineering viability for heterogeneous reservoirs.
(4) This work establishes a transferable framework for gel-based EOR, with future research prioritizing adaptive NGM injection (size, concentration optimization) and integration with auxiliary techniques (e.g., surfactant, thermal methods) for complex pore systems in ultra-deep or high-temperature reservoirs.

## 4. Mechanisms and Methods

### 4.1. Formation and Numerical Characterization of PFCs

#### 4.1.1. Formation Mechanism of PFCs

During prolonged water injection, injected water leaches and transports mudstone components, weakening interparticle cementation strength. This process promotes gradual pore-throat enlargement, reduces flow path tortuosity, and ultimately increases reservoir permeability by up to 50% in affected zones. Concurrently, crude oil adsorption capacity decreases while polar component adsorption increases. These changes shift rock wettability toward hydrophilicity, thereby enhancing fluid mobility (particularly aqueous phase). These coupled mechanisms establish high-connectivity preferential flow channels (PFCs) between injector and producer wells [17]. PFC development substantially exacerbates reservoir heterogeneity, causing injected water to bypass significant residual oil volumes. This constitutes the primary constraint on recovery enhancement during late-stage waterflooding.

#### 4.1.2. Numerical Modeling of PFC Evolution

Samier et al. [18,19] established that dynamic reservoir stress field alterations (e.g., rock compaction, fracture reactivation) during water injection constitute the primary driver of permeability evolution, which cannot be accurately captured by one-way coupling models. Therefore, this study employs the coupled CROCKTAB and CROCKTABW keywords to construct a multi-field coupling model. CROCKTAB defines complete loading and unloading paths, accounting for elastic and partial plastic rebound during pressure variations. Conversely, CROCKTABW defines a single loading path where rock compression and expansion occur without elastic rebound during pressure changes.

The selection of this dual-keyword coupling approach is grounded in three critical requirements:

(1) Physical fidelity requirement: Conventional one-way coupling models fail to capture the hysteresis effects in stress–permeability relationships during cyclic water injection. The dual-keyword approach dynamically couples rock deformation with aqueous-phase pressure evolution—essential for simulating irreversible PFC formation in long-term waterflooding scenarios.
(2) Field validation imperative: The CROCKTABW keyword explicitly links rock compaction to water injection operations (dominant in the Chang 6 reservoir), resolving the “static table limitation” of conventional geomechanical models. Field data confirm that the permeability changes correlate with water saturation increments.
(3) Numerical efficiency advantage: The coupled framework achieves accurate PFC characterization with <5% additional computation time versus decoupled approaches.

The CROCKTAB keyword in the CMG core defines the hysteresis relationship (loading and unloading path) between rock porosity and permeability as a function of effective stress (overlying formation stress–pore pressure). However the model is essentially a static table of rock mechanic ontogenetic relationships, which does not explicitly couple the hydrodynamic source of pore pressure variation with rock deformation. In real reservoirs, pressure changes are realized by fluid injection (pressurization) or extraction (depressurization), and fluid flow, phase distribution, and pressure propagation are the fundamental driving forces that drive changes in the effective stresses of compaction and trigger compaction and rebound. Therefore, in order to more realistically simulate the fluid–rock interaction in the process of water-driven development, the keyword CROCKTABW is also chosen in this paper. The core objectives are:

(1) To establish a coupling mechanism dominated by water-phase pressure: The water-phase fluid pressure is explicitly used as a direct input variable for calculating the effective stress change of rock grid cells. This allows the compaction or rebound response of the rock to be directly and dynamically bound to the flow, injection, or extraction processes of the water phase and the pressure field evolution in the model.
(2) Realize true fluid–solid coupling (partial coupling): With CROCKTABW, the change of rock properties no longer depends only on a preset, static pressure field or stress path, but responds in real time to local water-phase pressure changes generated by the water-driven process.
(3) Focus on water-driven dominated deformation processes: The model is explicitly instructed to focus on scenarios where the water phase is the main driver of pore pressure changes and rock deformation. This is especially applicable to oilfields where water injection is the main development method, and the main controlling factor of the rock mechanical response is the spatial and temporal distribution of water-phase pressure.
(4) Reflecting engineering reality: in oilfield sites, water injection operations directly affect reservoir pressure, which in turn changes the effective stress and leads to rock deformation. The choice of CROCKTABW is to reproduce the dynamic change of rock properties directly triggered by the waterflooding operation in the numerical model, so that the simulation results are more in line with the physical reality and engineering observations.

This choice resolves three critical gaps: hysteresis effects in stress–permeability relationships, waterflooding-specific deformation triggers, and computational efficiency for field-scale simulations.

#### 4.1.3. Characterization of PFC Dynamics via Coupled Simulation

Most geologic models are derived from initial logging curves obtained during the early development stage of oilfields. After prolonged waterflooding, quantitatively characterizing the preferential flow channels (PFCs) formed between injection and production wells under continuous aqueous-phase scouring constitutes a critical unresolved challenge. It is established that PFCs exhibit elevated flow diversion rates, high water saturation, and increased porosity and permeability relative to low-permeability strata. Consequently, porosity and permeability can be modeled as functions of water saturation [20].

(1) Pressure-induced temporal evolution of reservoir properties (CROCKTAB)

Permeability is governed by fluid porosity, which is influenced by pressure-dependent rock compaction during reservoir development. As pressure propagates through the fluid medium, permeability and porosity variations can be expressed as functions of pressure:

(3) $$\phi (P)=\phi_{i}\cdot m$$

(4) $$K(P)=K_{i}\cdot m$$

where *ϕ*_i_ denotes the initial porosity; *K*_i_ denotes the initial permeability; *ϕ*(P) is the porosity at pressure *P*; *K*(P) denotes the permeability at pressure *P*; and *m* is the multiplier at pressure *P*.

(2) Temporal variation of reservoir properties due to water saturation (CROCKTABW):

(5) $$\phi (\Delta S_{w})=\phi_{i}\cdot (1+n)$$

(6) $$K(\Delta S_{w})=K_{i}\cdot (1+n)$$

(7) $$\Delta S_{w}=S_{w}-S_{\mathrm{wi}}$$

where *S*_w_ is the dynamic water saturation; *S*_wi_ is the initial water saturation; ∆*S*_w_ is the incremental water saturation during displacement; *ϕ*(∆*S*_w_) is the dynamic porosity; *K*(∆*S*_w_) is the dynamic permeability; and *n* is the multiplier determined by ∆*S*_w_.

When a grid cell is subject to both CROCKTAB and CROCKTABW, the resultant porosity and permeability changes are:

(8) $$\phi (\Delta S_{w},P)=\phi_{i}\cdot \underset{m\geq 1\;\cap \;n\leq 1}{\max}(m,1+n)$$

(9) $$K\left(\Delta S_{w},P\right)=K_{i}\cdot \underset{m\geq 1\;\cap \;n\leq 1}{\max}(m,1+n)$$

### 4.2. Mechanisms of NGMs for Flooding and Profile Control

Wang et al. [21] demonstrated that conventional polymers exhibit peak oil-enhancement periods of only 0.5–1.5 years and effective durations of approximately 1.5–4.5 years, whereas NGMs achieve peak enhancement periods of 1.0–4.0 years and effective durations exceeding 8.0 years. Lin et al. [22] identified that microsphere migration involves surface adsorption, mechanical capture, hydrodynamic retention, and interparticle interactions. Their proposed multistage sub-nanosphere deep profile control technology for submarine fractured reservoirs significantly extends breakthrough times and mitigates gas channeling damage.

#### 4.2.1. Plugging Mechanism of NGMs

Lin et al. [23] utilized mercury intrusion porosimetry (constant-rate and constant-pressure methods) to establish that prolonged waterflooding enlarges pore throats, with larger throats dominating fluid seepage pathways. Jia et al. [24] further concluded that primary pore-throat radii expand after waterflooding, enhancing their seepage contribution, while pore-throat distributions remain largely unchanged. This confirms that pore-throat characteristics—rather than void space properties—govern reservoir-scale seepage behavior. Hua et al. [25] employed nuclear pore membrane filtration, sandpack displacement, core flooding, micromodel visualization, and capillary flow experiments to demonstrate that water-dispersed NGMs expand 3–6-fold in size, effectively plugging nuclear pore membranes. These systems selectively obstruct high-permeability zones and displace crude oil from low-permeability regions in parallel sandpacks. Cai et al. [26] derived an inverse relationship between the pore-throat ratio and permeability:

(10) $$K=\frac{\phi R_{\mathrm{pt}}^{2}r_{p}^{2}}{2(1+R_{\mathrm{pt}}^{4})(1+R_{\mathrm{pt}}^{2})\tau^{2}}$$

where *K* denotes the permeability; *ϕ* is the porosity; *R*_pt_ denotes the pore throat ratio; *r*_p_ denotes the pore-throat radius; and *τ* denotes the pore tortuosity.

#### 4.2.2. Fluid Diversion Mechanism

Prior studies have confirmed that partial or complete pore-throat blockage in high-conductivity channels elevates viscosity or reduces permeability, thereby increasing the pressure gradient and diverting flow toward low-permeability strata. Li et al. [27] simulated formation fractures using single-sandpack glass capillaries, revealing that diminished deep-reservoir capillary forces reduce aqueous-phase seepage velocity. Under such conditions, particle Brownian motion becomes significant, further suppressing the water-phase permeability and enabling the gradual conversion of injected water into formation energy. This facilitates deep-reservoir energy buildup and fluid diversion. Li et al. [28] and Wang et al. [29] mathematically established that NGM deposition significantly increases the pore-wall surface area while reducing the capillary channel volume and elevating the specific surface area. They identified nanoscale polymer microsphere adsorption on pore surfaces as the fundamental permeability-alteration mechanism. Jiang et al. [30] experimentally observed solid–liquid interfacial adsorption during microsphere transport through pore channels; wall-adhered microspheres reduce effective pore radii, enabling flow steering under incomplete blockage. Wang et al. [31] combined experiments with SEM imaging to demonstrate that adsorbed polymer microspheres reduce pore-throat dimensions while increasing the specific surface area, thereby lowering high-permeability-layer permeability and inducing fluid diversion.

#### 4.2.3. Numerical Modeling of NGM Flooding Mechanisms

Key parameters (concentration, size, adsorption coefficients) were empirically optimized through laboratory core-flooding and validated in field applications, ensuring reliability without additional sensitivity analysis. In ongoing experimental and simulation studies aimed at optimizing NGM concentration and properties, preliminary findings indicate that a 1% concentration offers a favorable balance between performance and cost, though this is subject to reservoir-specific conditions [32,33,34,35]. Relevant research findings reveal concentration-dependent tradeoffs. Specifically, a 1% concentration strikes a balance between plugging efficiency (achieving permeability reduction of over 30%) and injectivity (keeping the pressure rise under 20%). The size range of nano-gel microspheres is determined to be 100~500 nm via pore-throat matching using mercury intrusion porosimetry. Among them, smaller microspheres (100~200 nm) can enhance access to low-permeability zones, but require higher concentrations to achieve equivalent PFC blockage. Injection strategies with adaptive concentration adjustments could further enhance NGM flooding efficiency. These insights have been incorporated into our numerical model to guide more accurate simulations of NGM flooding mechanisms.

The resistance coefficient *F*_r_ and residual resistance coefficient *F*_rr_ serve as critical indices for quantifying elastic microsphere plugging performance [36,37]. Zhou et al. [38] injected diverse fluids into formations and computed these coefficients using Hall plot slope variations across injection stages to assess permeability reduction in high-permeability zones. For the current simulations, we assume that no significant degradation of NGMs occurs within the 10-year timeframe based on their demonstrated thermal stability (>90 °C) and salinity tolerance (>20,000 ppm), though degradation kinetics will be incorporated in future models as the experimental data mature. These coefficients are derivable via Hall curve analysis during three key phases: the initial waterflooding, NGM injection, and post-flushing stages.

Based on the above theory, the absolute permeability within NGM-influenced regions is calculated as:

(11) $$\bar{K}=\frac{K(\Delta S_{w},P)}{R_{K}}$$

where $R_{K}=1+(F_{\mathrm{rr}}-1)\cdot \frac{AD}{ADMAXT}$ and $F_{\mathrm{rr}}=\frac{\lambda_{w}}{\lambda_{w}^{\prime }}=\frac{k_{w}/\mu_{w}}{k_{w}^{\prime }/\mu_{w}^{\prime }}$ represent the resistance and residual resistance coefficients, respectively; *AD* is the cumulative polymer adsorption per unit rock volume; *ADMAXT* is the maximum polymer adsorption per unit rock volume; $\bar{K}$ is the absolute permeability in NGM-affected zones; *λ*_w_ and *λ*′_w_ denote the pre- and post-NGM injection aqueous-phase mobility; *k*_w_ and *k*′_w_ are the pre- and post-NGM effective aqueous-phase permeabilities; and *μ*_w_ and *μ*′_w_ are the pre- and post-NGM aqueous-phase viscosities.

Then, the absolute permeability within NGM-influenced regions is calculated as:

(12) $$\bar{K}=\frac{K(\Delta S_{w},P)}{1+\left(\frac{k_{w}/\mu_{w}}{k_{w}^{\prime }/\mu_{w}^{\prime }}-1\right)\cdot \frac{AD}{ADMAXT}}$$

Thus, the effective permeability for any fluid phase ‘a’ in NGM-swept zones is:

(13) $$k_{a}=\bar{K}\cdot k_{\mathrm{ra}}=\frac{K(\Delta S_{w},P)\cdot k_{\mathrm{ra}}}{1+\left(\frac{k_{w}/\mu_{w}}{k_{w}^{\prime }/\mu_{w}^{\prime }}-1\right)\cdot \frac{AD}{ADMAXT}}$$

where *K*_a_ is the phase-effective permeability and *K*_ra_ is the phase-relative permeability.

Incorporating Equation (8) for dual CROCKTAB and CROCKTABW effects yields the dynamic effective permeability:

(14) $$k_{a}=\bar{K}\cdot k_{\mathrm{ra}}=\frac{k_{i}k_{\mathrm{ra}}\underset{m\geq 1\;\cap \;n\leq 1}{\max}(m,1+n)}{1+\left(\frac{k_{w}/\mu_{w}}{k_{w}^{\prime }/\mu_{w}^{\prime }}-1\right)\cdot \frac{AD}{ADMAXT}}$$

## Figures and Tables

**Table 1:** Physical properties of the reservoir simulation model.

Reservoir Properties	Value
Depth to oil layer midpoint (m)	1210
Reference pressure (MPa)	9.62
Crude oil formation volume factor (rm^3^/sm^3^)	1.206
Crude oil compressibility (MPa^−1^)	9.816 × 10^−5^
Crude oil density (kg/m^3^)	841
Crude oil viscosity (mPa·s)	2.62
Water compressibility (MPa^−1^)	5.0 × 10^−5^
Water viscosity (mPa·s)	0.5
Rock compressibility (MPa^−1^)	7.135 × 10^−5^

**Table 2:** Property evolution in high-permeability layer during waterflooding stages.

	Initial Development Stage	Waterless Oil Recovery Stage	Water Breakthrough Stage	High Water Cut Stage	
Porosity	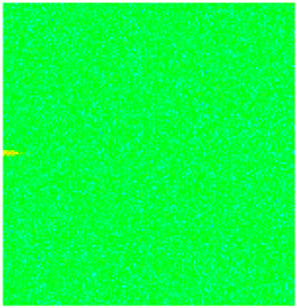	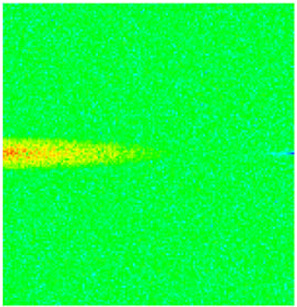	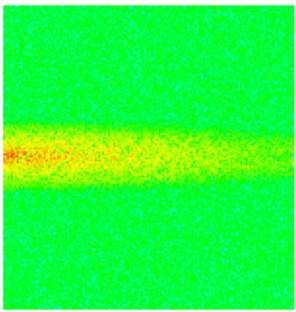	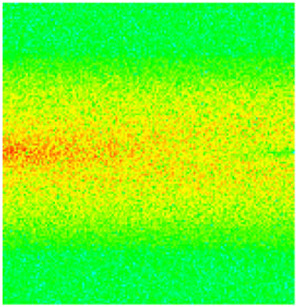	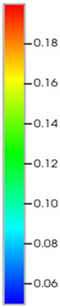
Permeability	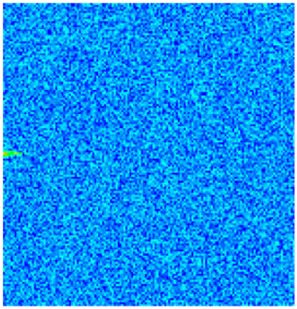	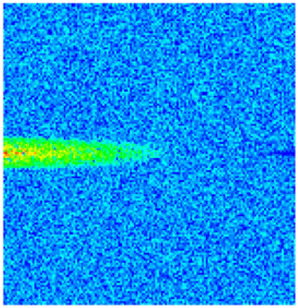	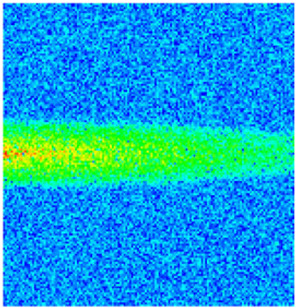	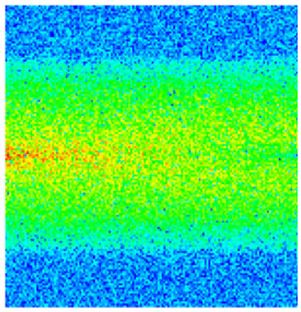	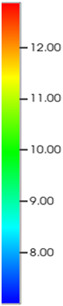
Oil saturation	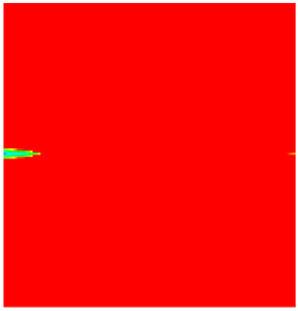	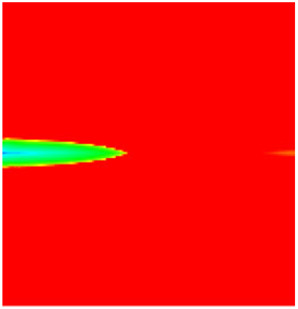	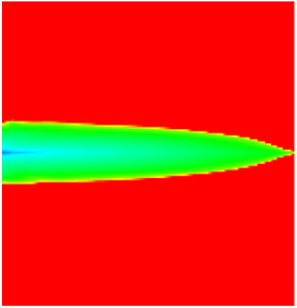	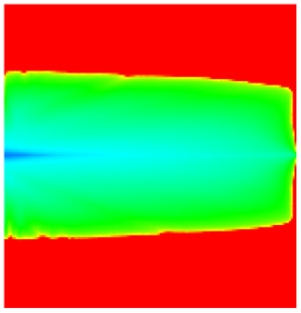	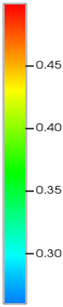

**Table 3:** Property evolution in low-permeability layer during waterflooding stages.

	Initial Development Stage	Waterless Oil Recovery Stage	Water Breakthrough Stage	High Water Cut Stage	
Porosity	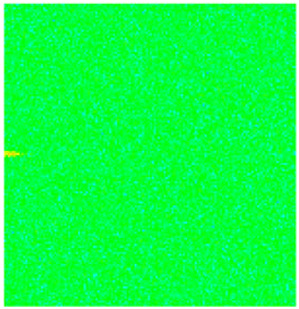	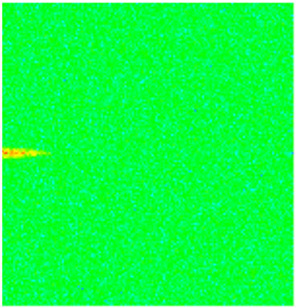	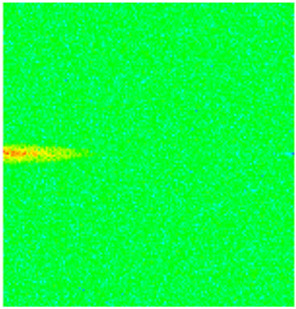	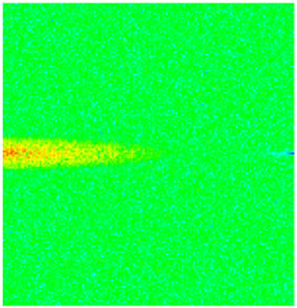	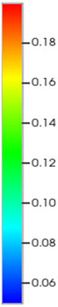
Permeability	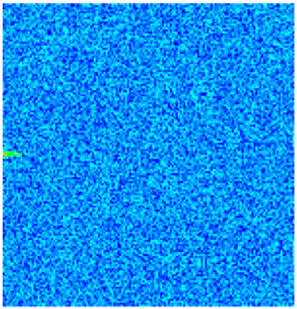	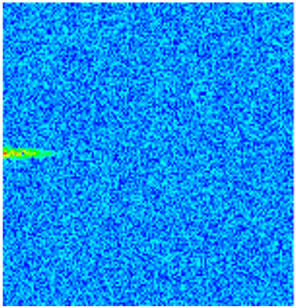	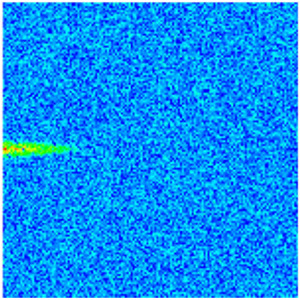	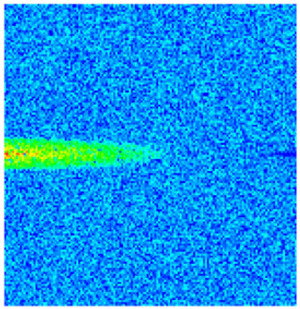	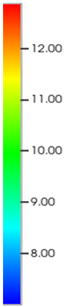
Oil saturation	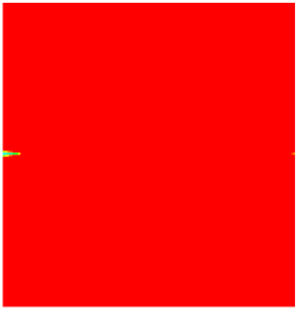	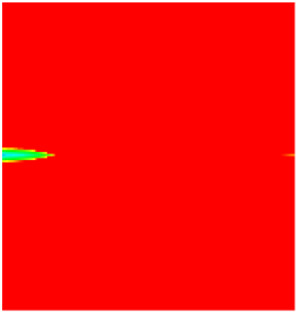	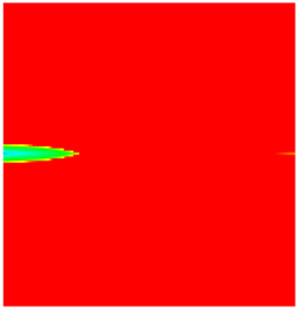	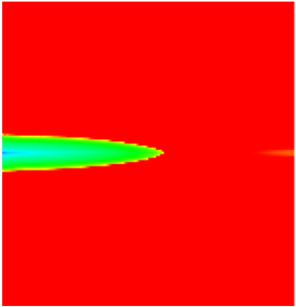	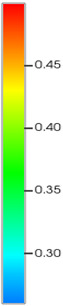

**Table 4:** Longitudinal profile evolution along injector–producer connection line during waterflooding stages.

	Initial Development Stage	Waterless Oil Recovery Stage	Water Breakthrough Stage	High Water Cut Stage
Porosity	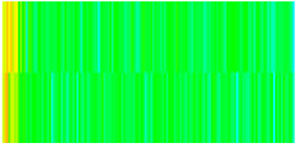	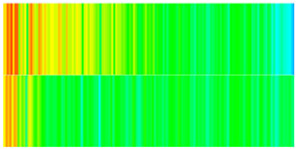	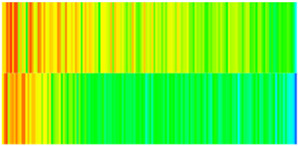	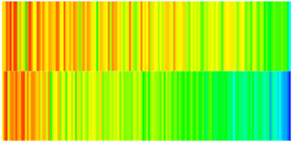
Permeability	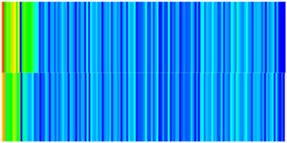	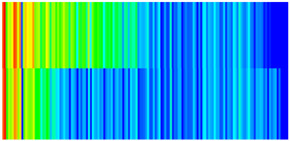	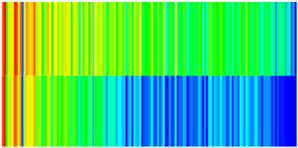	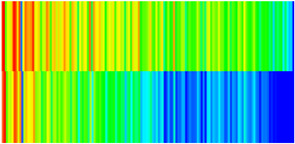
Oil saturation	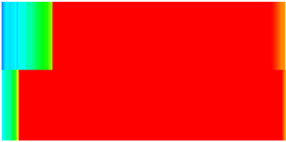	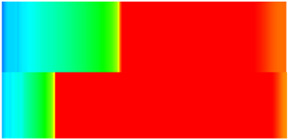	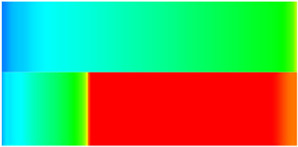	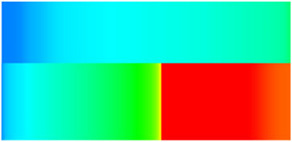

**Table 5:** Characteristics of dynamic changes in physical properties of relatively high permeability layers during NGM flooding after waterflooding.

	Early Stage of NGMs	Middle Stage of NGMs	Late Stage of NGMs	Final Stage of NGMs	
Porosity	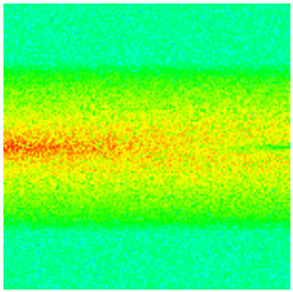	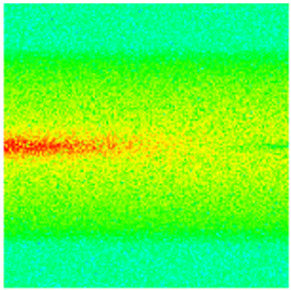	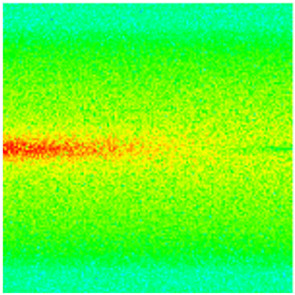	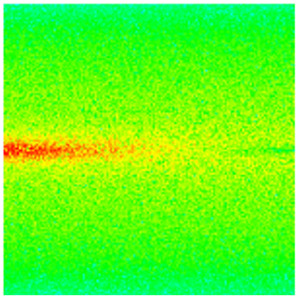	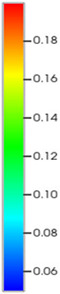
Permeability	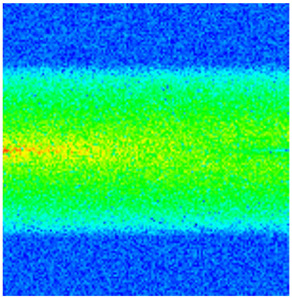	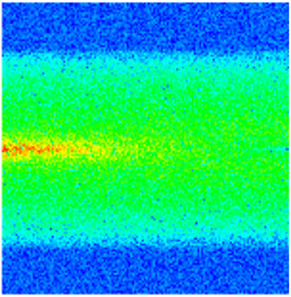	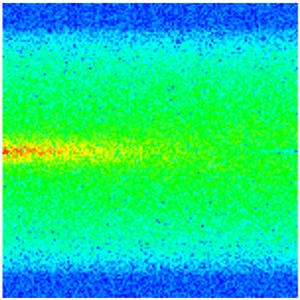	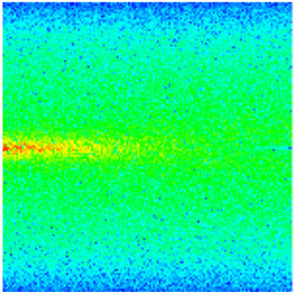	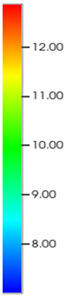
Oil saturation	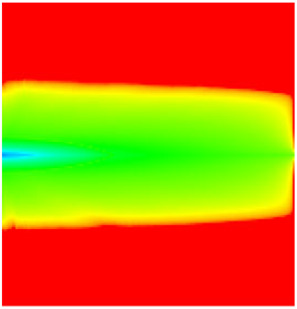	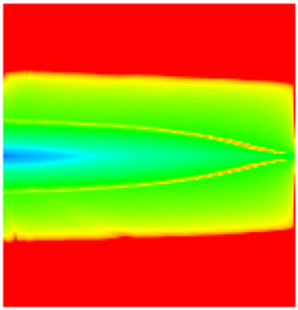	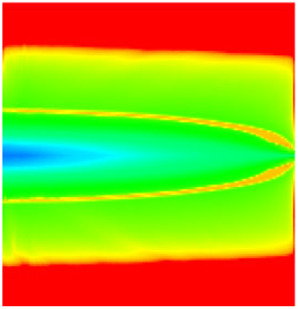	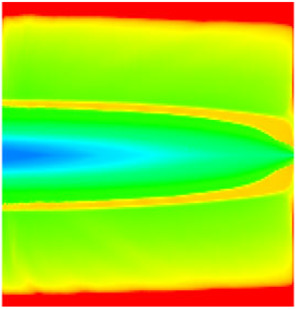	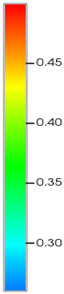

**Table 6:** Characteristics of dynamic changes in physical properties of relatively low permeability layers during NGM flooding after waterflooding.

	Early Stage of NGMs	Middle Stage of NGMs	Late Stage of NGMs	Final Stage of NGMs	
Porosity	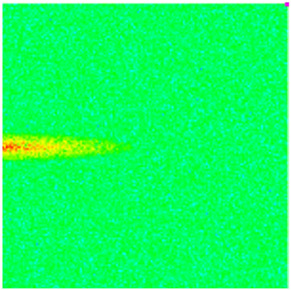	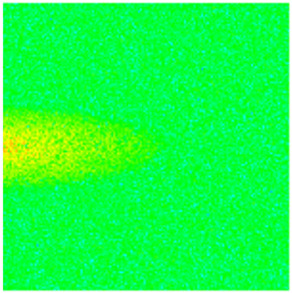	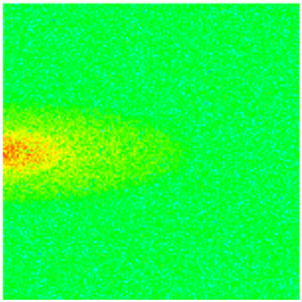	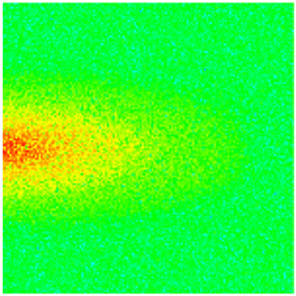	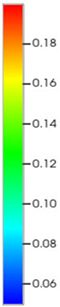
Permeability	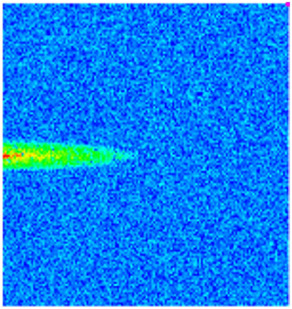	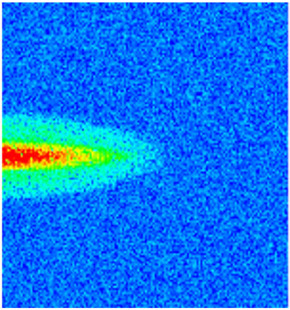	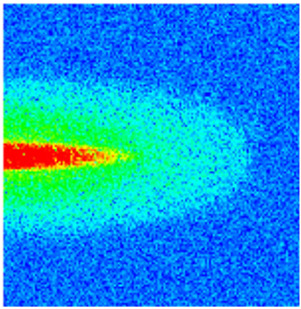	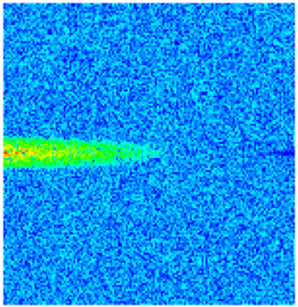	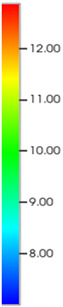
Oil saturation	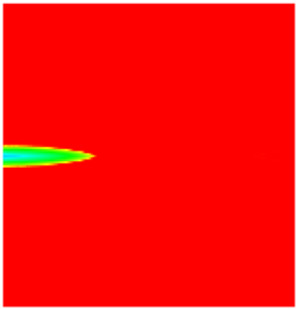	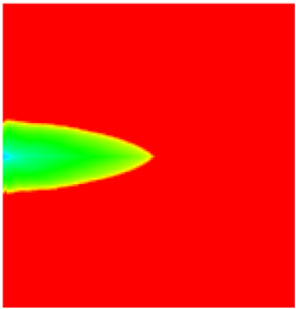	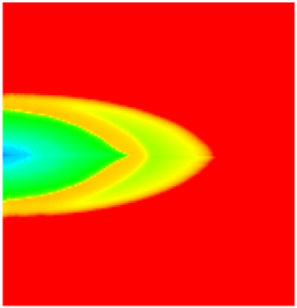	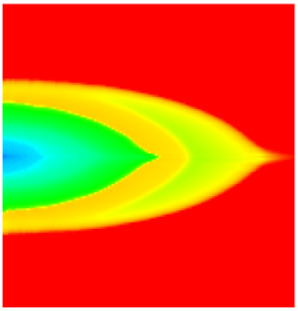	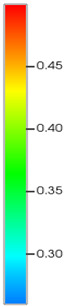

**Table 7:** Characteristics of dynamic changes in physical properties of longitudinal profiles between injection and production well during NGM flooding after waterflooding.

	Early Stage of NGMs	Middle Stage of NGMs	Late Stage of NGMs	Final Stage of NGMs
Porosity	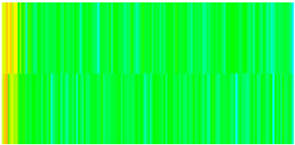	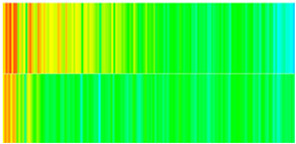	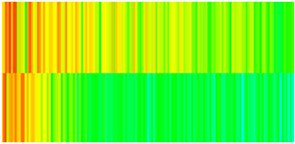	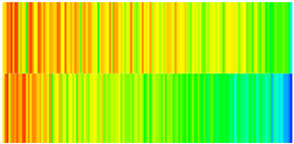
Permeability	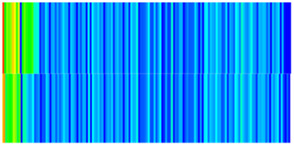	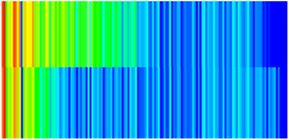	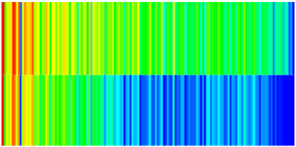	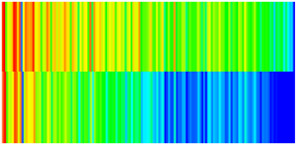
Oil saturation	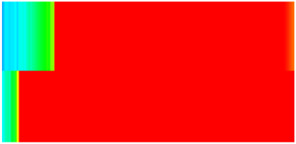	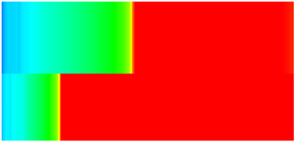	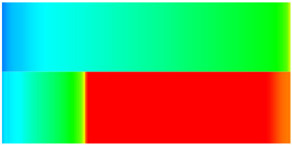	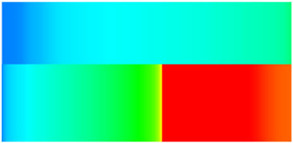

## Data Availability

The raw data supporting the conclusions of this article will be made available by the authors on request.
